# Supplementary material for: Investigating the influence of physiologically relevant hydrostatic pressure on CHO cell batch culture
Source: Sci Rep. 2021 Jan 8;11:162. doi: 10.1038/s41598-020-80576-8 (PMC7794228; doi:10.1038/s41598-020-80576-8)
Supplement: Supplementary file 1 — Supplementary Information 1. [file 41598_2020_80576_MOESM1_ESM.zip › S1/IgG/18Dec 4th round 5%CO2/SS1.pdf]

===

Acq. Operator	: SYSTEM	Seq. Line :	8
Sample Operator	: SYSTEM		
Acq. Instrument	: HPLC-0039	Location :	6
Injection Date	: 12/18/2018 12:16:25 PM	Inj :	1
		Inj Volume :	100.000 µl
Sequence File	: C:\Users\Public\Documents\ChemStation\1\Data\CHO_IgG 2018-12-18 11-44-16 \CHO_IgG.S		
Method	: C:\Users\Public\Documents\ChemStation\1\Data\CHO_IgG 2018-12-18 11-44-16 \CHO_IGG_LC.M (Sequence Method)		
Last changed	: 12/18/2018 11:44:20 AM by SYSTEM		

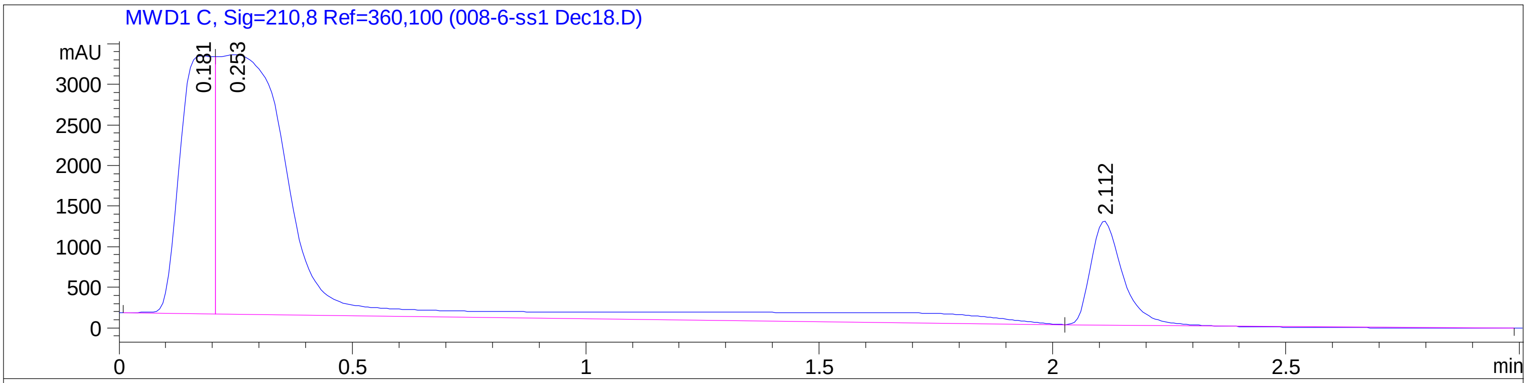

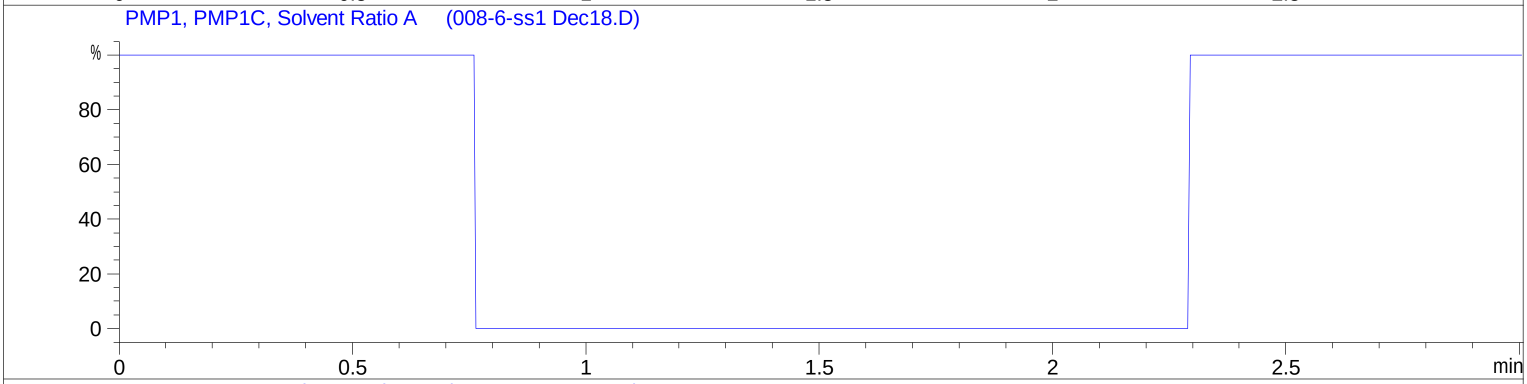

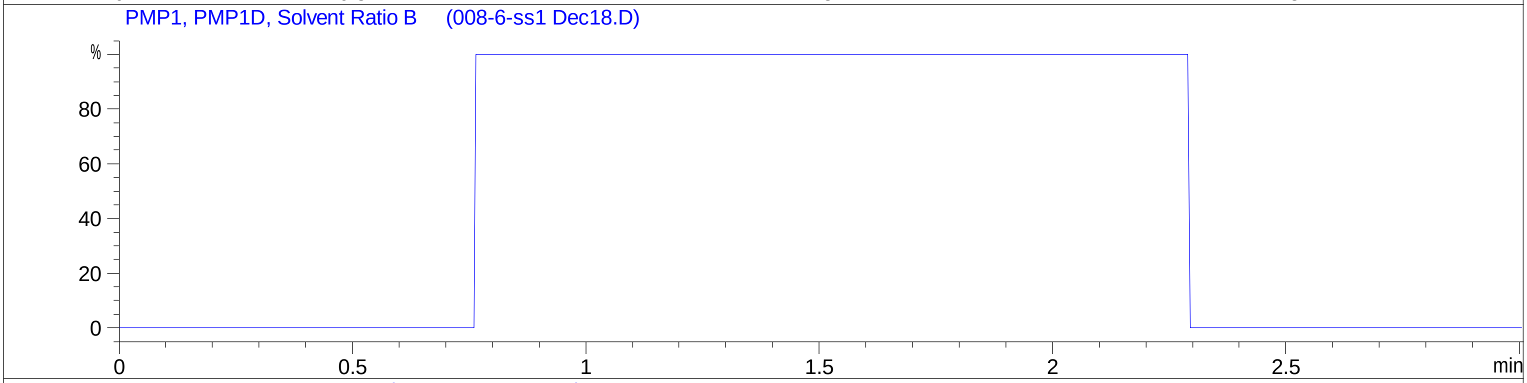

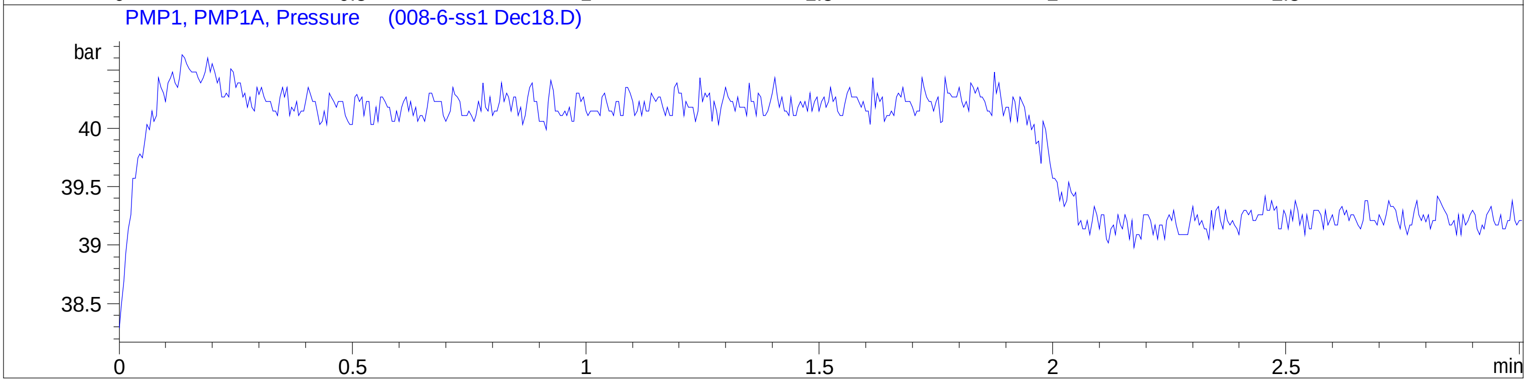

===

Area Percent Report

===

Sorted By : Signal
Multiplier : 1.0000
Dilution : 1.0000
Use Multiplier & Dilution Factor with ISTDs

Signal 1: MWD1 C, Sig=210,8 Ref=360,100

Peak #	RetTime [min]	Type	Width [min]	Area [mAU*s]	Height [mAU]	Area %
1	0.181	BV	0.0760	1.52373e4	3175.86133	24.9866
2	0.253	VV	0.1855	3.98756e4	3197.43579	65.3895
3	2.112	VB	0.0694	5868.78369	1281.53784	9.6238

Totals : 6.09817e4 7654.83496

===

*** End of Report ***
